# Effects of Growth Phase and Temperature on **σ** ^B^ Activity within a *Listeria monocytogenes* Population: Evidence for RsbV-Independent Activation of **σ** ^B^ at Refrigeration Temperatures

**DOI:** 10.1155/2014/641647

**Published:** 2014-03-05

**Authors:** Marta Utratna, Eoin Cosgrave, Claas Baustian, Rhodri H. Ceredig, Conor P. O'Byrne

**Affiliations:** ^1^Bacterial Stress Response Group, Microbiology, School of Natural Sciences, National University of Ireland, Galway, Ireland; ^2^Waters Corporation, Milford, MA 01757, USA; ^3^Regenerative Medicine Institute (REMEDI), National Centre for Biomedical Engineering Science, National University of Ireland, University Road, Galway, Ireland

## Abstract

The alternative sigma factor *σ* ^B^ of *Listeria monocytogenes *is responsible for regulating the transcription of many of the genes necessary for adaptation to both food-related stresses and to conditions found within the gastrointestinal tract of the host. The present study sought to investigate the influence of growth phase and temperature on the activation of *σ* ^B^ within populations of *L*. *monocytogenes* EGD-e wild-type, Δ*sigB, *and Δ*rsbV *throughout growth at both 4°C and 37°C, using a reporter fusion that couples expression of EGFP to the strongly *σ* ^B^-dependent promoter of *lmo*2230. A similar *σ* ^B^ activation pattern within the population was observed in wt-*egfp *at both temperatures, with the highest induction of *σ* ^B^ occurring in the early exponential phase of growth when the fluorescent population rapidly increased, eventually reaching the maximum in early stationary phase. Interestingly, induction of *σ* ^B^ activity was heterogeneous, with only a proportion of the cells in the wt-*egfp *population being fluorescent above the background autofluorescence level. Moreover, significant RsbV-independent activation of *σ* ^B^ was observed during growth at 4°C. This result suggests that an alternative route to *σ* ^B^ activation exists in the absence of RsbV, a finding that is not explained by the current model for *σ* ^B^ regulation.

## 1. Introduction

The ability of *Listeria monocytogenes* to cause foodborne listeriosis depends on a multifaceted stress response that is activated under conditions related to food processing including high concentrations of salt, acidic pH, limited oxygen, antimicrobial agents, and a wide range of temperatures from −0.4 to 45°C [1]. The remarkable adaptability of this bacterium is partly modulated by transcriptional regulators that tailor gene expression to the conditions encountered, with a pivotal role being played by sigma factors that target RNA polymerase to specific promoter sequences [2]. The alternative sigma factor, sigma B (*σ* ^B^), first identified in *L. monocytogenes* based on homology to the general stress response sigma factor from the nonpathogenic bacterium *Bacillus subtilis* [3, 4] coordinates the response to a range of stresses as evidenced by the pleiotropic phenotypes associated with a *sigB* deletion (reviewed by [5]). A core set of *σ* ^B^-dependent genes (*σ* ^B^ regulon) has been described in *L. monocytogenes* and shown to be upregulated in response to a range of conditions including osmotic stress, cold shock, heat shock, acid stress, during stationary phase of growth, and under conditions encountered in gastrointestinal tract [3, 6–9]. There is significant overlap between the PrfA virulence regulon and the *σ* ^B^ regulon [10] with evidence that *σ* ^B^ may even modulate PrfA activity at the intracellular stage of infection [11]. Thus *σ* ^B^ plays important roles in both virulence and in the general response to stress, which makes understanding its regulation essential for future strategies that aimed at controlling this pathogen in the food chain and within the host.

The current model of *σ* ^B^ activation in *L. monocytogenes *is based on the high level of similarity of the *sigB* operon to that from *B. subtilis* (*rsbR-rsbS-rsbT-rsbU-rsbV-rsbW-sigB-rsbX*) and it suggests posttranslational regulation of *σ* ^B^ activity together with *σ* ^B^ autoregulation at the transcriptional level [12]. In the absence of stress stimuli *σ* ^B^ interacts with an antisigma factor, RsbW, which renders it unavailable for interaction with RNA polymerase. The dephosphorylation of the anti-anti-sigma factor RsbV, which occurs in response to stress, is catalysed by the protein phosphatase RsbU. This renders RsbV capable of interacting with RsbW, which in turn liberates *σ* ^B^, allowing it to participate in transcription. RsbU activity is in turn regulated through an interaction with RsbT, whose availability is determined by its association with a high molecular weight (~2 MDa) stress sensing complex called a “stressosome”. *B. subtilis* environmental (physical and chemical) stresses influence *σ* ^B^ activity in this way but energy-related stresses are transduced by an alternative phosphatase called RsbP. Since no homologue of RsbP is encoded in the genome of *L. monocytogenes* both environmental and energy stresses are proposed to be transmitted through RsbU [13, 14], but in both organisms the initial stress sensing mechanism remains to be elucidated.

Several studies have investigated the role of *σ* ^B^ in allowing *L. monocytogenes* to grow at low temperatures. However studies addressing the effects of a *sigB* deletion on the phenotypic characteristics of *L. monocytogenes* at low temperature have reported conflicting observations. A Δ*sigB* strain of *L. monocytogenes* 10403S had reduced growth in a defined medium (DM) at 8°C [3] and *L. monocytogenes* EGD-e Δ*sigB* was reported to be sensitive to freeze-thaw cycles [15]. In contrast, a Δ*sigB* derivative of *L. monocytogenes* EGD did not show impaired growth in DM at 3°C [16] and *L. monocytogenes* 10403S Δ*sigB* had a similar growth pattern to the wild-type when grown at 4°C in BHI over 12 days [17, 18]. The available evidence is also unclear on the question of whether of *σ* ^B^ activity is elevated during growth at low temperatures. Transcription of the autoregulated *sigB* operon is induced at cold temperatures suggesting that *σ* ^B^ activity is elevated during low temperature growth [3, 19]. The promoter of the *opuC* operon, which encodes a compatible solute uptake system known to be regulated by *σ* ^B^ [20–22], has also been used to look at *σ* ^B^ activity at low temperatures. One study reported that *opuCA *transcript levels are unaffected during temperature downshift or growth at 4°C [17], while another study observed that *opuCA* transcription is induced after temperature downshift [13]. However, the presence of a *σ* ^A^-dependent promoter upstream from *opuCA* makes interpretation of these results more difficult [17]. Thus the uncertainty in the literature regarding the role and regulation of *σ* ^B^ during cold adaptation made it important to investigate this question further.

To clarify the role and activity of *σ* ^B^ during adaptation to low temperature growth we have monitored *σ* ^B^ activity during prolonged growth at 4°C in comparison to a culture growing at 37°C. *σ* ^B^ activity was monitored by measuring the expression of two genes known to be under direct *σ* ^B^ control, *opuCA,* and *lmo2230,* which encodes a putative arsenate reductase [20, 23]. Polyclonal antibodies were used to detect the OpuCA protein while *lmo2230* expression was monitored using an EGFP (enhanced green fluorescent protein) reporter fusion to the strongly *σ* ^B^-dependent promoter of the *lmo2230 *gene [22, 24]. Fluorescence measurements were made using flow cytometry throughout growth in wild-type, Δ*sigB* and Δ*rsbV *backgrounds. The measurements revealed heterogeneous activation of *σ* ^B^ within growing populations of cells, with increased activation evident as cells progressed through exponential phase, reaching maximum activation in stationary phase. Similar results were observed at both growth temperatures suggesting that *σ* ^B^ activity was not increased by reduced temperatures *per se*. The study provides important new insights into the temporal and population-related parameters that modulate the activity of sigma B in *L. monocytogenes*.

## 2. Materials and Methods 

### 2.1. Growth Conditions

*L. monocytogenes* EGD-e strains used in this study are listed in Table 1. For growth and flow cytometry (FCM) experiments at 37°C overnight cultures (16 h) were inoculated into 25 mL of sterile Brain Heart Infusion (BHI) broth (LabM) in 250 mL flasks to give a starting OD_600_ = 0.05. OD_600_ readings that were taken from rotary (180 rpm) shaken cultures at 45 min intervals over 7 hours and samples of cells were taken for FCM and fixed as previously described [24]. Growth at 4°C was also monitored from a starting OD_600_ = 0.05. Stationary culture cells grown in BHI at 37°C were inoculated into 200 mL of sterile BHI in 2 L flasks and gently agitated (30 rpm) on the rocker (Stuart See-Saw Rocker SSL4) at 4°C. Samples were taken daily over 12 days for monitoring OD_600_ and at 3-day intervals for FCM.

### 2.2. Protein Extraction

For determining levels of *σ* ^B^ bacterial cultures were grown in 50 mL of BHI for 3 h at 37°C. Then 1 mL of culture was transferred at 60 min intervals into a tube containing chloramphenicol at a final concentration of 10 *μ*g mL^−1^ to prevent further protein translation during the sample preparation steps. Samples were stored on ice until the completion of the experiment. Each 1 mL culture was then centrifuged at 12,000 g for 10 min to pellet bacterial cells. The supernatant was discarded and each cell pellet was resuspended in 100 *μ*L of BugBuster cell lysis reagent (Novagen, USA) containing 1% (v/v) DNaseI, 1% (v/v) Halt Protease Inhibitor Cocktail, and 1% (w/v) Lysozyme (Sigma, USA). Each cell suspension was then incubated at 37°C for 1 h with agitation. The resulting cell lysates were centrifuged at 5,000 g for 10 min to remove insoluble material. For OpuCA levels large-scale protein extraction was carried out by growing bacterial cultures at 37°C with a volume of 500 mL and removing 50 mL of cultures at 50 min intervals into a tube containing chloramphenicol at a final concentration of 10 *μ*g mL^−1^. A disruption of cells was accomplished by sonication and protein extraction was performed as previously described [6]. For EGFP and RsbW levels proteins were extracted from 100 mL of stationary cultures grown for 12 days at 4°C using the sonication-based method with a slight modification; cells were fixed in 1 : 1 volume of ice cold 1 : 1 (v/v) methanol/ethanol mixture for 10 min at −20°C before centrifugation. The concentrations of protein extracts were determined by the RC DC Protein Assay Kit (BioRad). The purified protein extracts were stored at −20°C until required.

### 2.3. Polyclonal Antisera Generation

DNA sequences corresponding to *rsbW* and *sigB* were PCR amplified from *L*. *monocytogenes* EGD-e using primers listed in Table 2. Each gene fragment was then cloned into pET101D with T7 promoter to include a 3′ polyhistidine tag for downstream purification requirements, yielding pEC02 and pEC03 which were transformed into *E. coli* BL21 (DE3). Cultures of *E. coli* harbouring either *rsbW-his6* or *sigB-his6* were grown in LB broth supplemented with 100 *μ*g mL^−1^ ampicillin and induced for overexpression by addition of 1 mM IPTG at approximately OD_600_ of 0.5. Cells were grown for 4 h following induction and collected by centrifugation at 10,000 g, washed with sterile media, and lysed with Bugbuster (Novagen, USA) containing 0.1% (v/v) DNaseI. The bacterial cell lysate was partitioned between soluble and insoluble material by centrifugation at 10,000 g for 10 min. Recombinant proteins present as inclusion bodies were purified from the insoluble fraction by Ni-NTA affinity chromatography using buffers supplemented with 8 M urea. RsbW-His6 and SigB-His6 were subsequently purified and prepared as 2 mg mL^−1^ stocks for immunization carried out by Fusion Antibodies (Belfast). For each protein two NZW rabbits were injected at several sites on each animal with 1 mL of each recombinant protein stock solution supplemented with Freund's adjuvant. 35 days following boosting serum from individual rabbits was tested for specificity against each target antigen. Approximately 5 months after-immunization antiserum against each antigen was collected. The rabbit anti-RsbW and anti-SigB IgG from each rabbit serum was isolated using protein A chromatography. Each IgG preparation was tested for specificity against target antigens using Western blotting.

### 2.4. Western Blotting

Western blotting analyses were performed using polyclonal antisera (1°Ab) developed in rabbits against *σ* ^B^ and RsbW (this study) or in chickens against OpuCA [22] and against GFP (Abcam) with commercial secondary antibodies (2°Ab) HRP-conjugated anti-rabbit or anti-chicken (Promega) at appropriate dilutions (Table 3) in 3% w/v skim milk. Protein extracts were normalized to 5 mg mL^−1^ total protein concentrations and 10 *μ*L of these samples was separated by SDS-PAGE and transferred to a nitrocellulose membrane. Membranes were blocked for 60 min in 3% (w/v) skim milk at room temperature and incubated overnight at 4°C with an appropriate 1°Ab. Incubations with 2°Ab were performed at room temperature for 60 min. Three ten-minute-long washing steps with Tween20 (Promega) diluted in PBS (Table 3) followed by 10 min in PBS were performed after each incubation with 1°Ab and 2°Ab. Blots were viewed with a chemiluminescent substrate (SuperSignal West Pico Chemiluminescent Kit, Pierce) using a light sensitive film (Amersham Hyperfilm ECL, GE Healthcare) or FluorChem Imager (Alpha Innotech Corp) and FluorChem IS-8900 software.

### 2.5. Flow Cytometry

FCM compared fluorescence of the fusion strains (wt*-egfp*, Δ*sigB-egfp*, Δ*rsbV-egfp*) to one another. The parent strains (wt, Δ*sigB*, Δ*rsbV*) were used to determine the level of autofluorescence and to define the EGFP gate above it. Analyses were performed with a BD Accuri C6 flow cytometer (Accuri Cytometers, Inc.) on 100 *μ*L of the fixed and PBS-suspended cells from 96 round bottom plates (Sarstedt). The 488 nm blue laser excitation, FL1 533/30 nm (e.g. FITC/GFP) emission channel, and 66 *μ*L/min flow rate were used. Each sample was performed in biological triplicate with duplicate analyses in each replication. A minimum of 100,000 events for each sample were recorded and processed with BD CFlow Software to determine % of fluorescent population and mean fluorescence values.

### 2.6. Visualization of the Fluorescence with Microscopy

One mL aliquots of bacterial cells was harvested with benchtop centrifuge at room temperature and subsequently resuspended in 50 *μ*L of sterile PBS. Five *μ*L of the suspension was smeared on microscope slides for visualization with Nikon Eclipse E600 microscope with a CCD camera attached using the B-2A filter and 1/8 Neutral Density (ND8) filter set. Images were recorded and processed for publication with ImageJ 1.44 software.

## 3. Results

### 3.1. *σ* ^B^ Activity Is Not Correlated with Cellular Levels of *σ* ^B^ Protein

**Figure 1:**
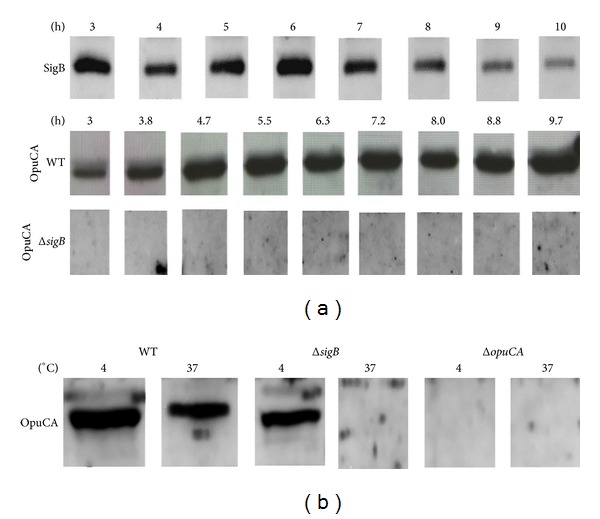
Levels of *σ* ^B^ and OpuCA at selected points of growth at 37°C and 4°C. (a) *σ* ^B^ levels were determined with Western blotting and rabbit polyclonal anti-*σ* ^B^ antibodies in *L. monocytogenes* wild-type grown in BHI at 37°C at 60 min intervals from mid-exponential to stationary phase. OpuCA levels were monitored with Western blotting and chicken polyclonal anti-OpuCA antibodies in *L. monocytogenes* wild-type and Δ*sigB *grown in BHI at 37°C at 50 min intervals from mid-exponential to stationary phase. (b) Levels of OpuCA were determined in *L. monocytogenes* wild-type, Δ*sigB, *and Δ*opuCA *grown in BHI up to exponential phase (OD_600_ = 0.6) at 4°C in comparison to 37°C.

Stationary phase is known to induce *σ* ^B^-dependent gene expression [3, 25] and *sigB* itself is known to be autoregulated at the transcriptional level. To determine if the levels of *σ* ^B^ changed during growth at 37°C Western blotting was performed on protein extracts taken at 60 min intervals from early exponential to stationary phase using anti-*σ* ^B^ polyclonal antibodies. Surprisingly, the highest levels of *σ* ^B^ were observed in the exponential phase of growth and *σ* ^B^ levels decreased when the cells entered stationary phase (Figure 1(a)). However, the model for *σ* ^B^ regulation established in *B. subtilis* suggests that *σ* ^B^ activity in *L. monocytogenes* is modulated primarily at the posttranslational level by a partner switching mechanism [26]. Thus availability of *σ* ^B^ rather than *σ* ^B^ levels determines its involvement in transcription. To determine when *σ* ^B^ was active during growth in *L. monocytogenes* the expression of the *σ* ^B^-dependent gene *opuCA* was measured at protein level. OpuCA levels were shown to be significantly lower in exponential phase of growth than observed in stationary phase in *L. monocytogenes* EGD-e grown at 37°C (Figure 1(a)). Furthermore, OpuCA was not detectable in the Δ*sigB* background at any point of growth confirming *σ* ^B^-dependent expression of *opuCA* under the conditions tested. During growth at 4°C OpuCA levels were measured in exponential phase and compared to the corresponding extracts from 37°C growth. OpuCA levels were increased at 4°C in comparison to 37°C (Figure 1(b)). However, significant levels of OpuCA were detected in the Δ*sigB* background suggesting that *opuCA *is expressed, at least partly, in a *σ* ^B^-independent manner at 4°C, a finding that is consistent with an earlier transcriptomic study [17]. Thus OpuCA levels are not a reliable indicator of *σ* ^B^ activity during growth at refrigeration temperatures.

### 3.2. *σ* ^B^ Is Activated during Exponential Growth at Both 37°C and 4°C

**Figure 2:**
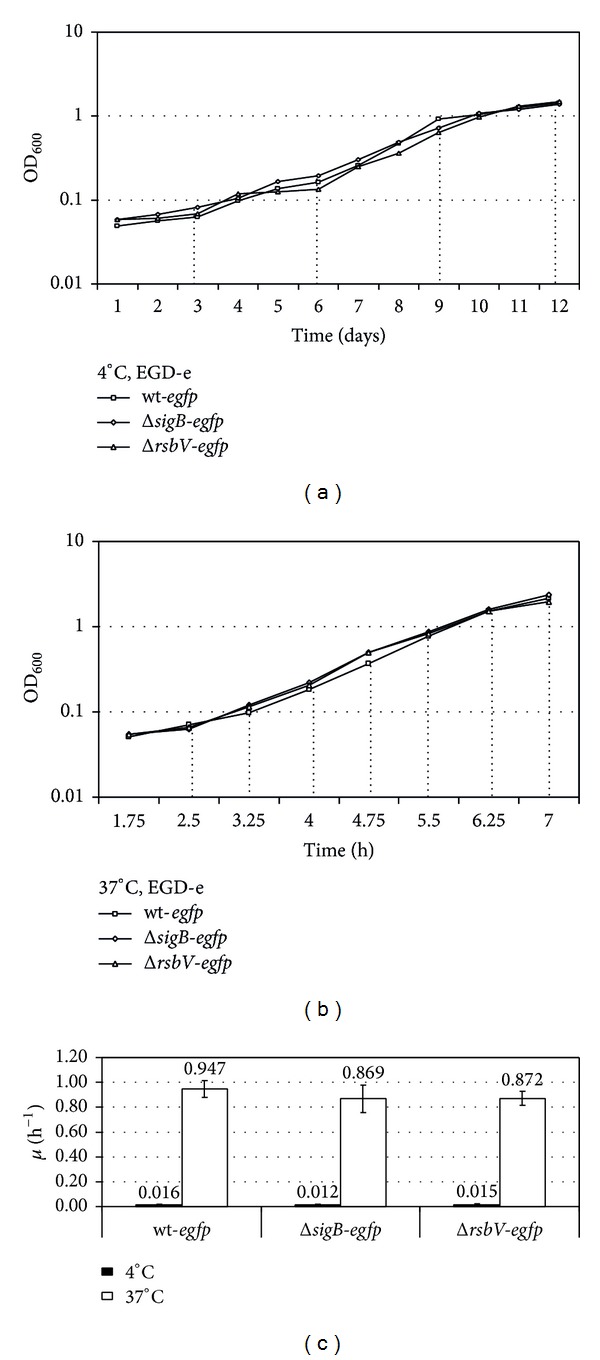
Growth of fusion strains at 4°C and 37°C. Strains of *L. monocytogenes *EGD-e wild-type, Δ*sigB,* and Δ*rsbV* containing P_*lmo2230*_::*egfp* fusion were grown at (a) 4°C and at (b) 37°C in BHI. Time points when samples of cells were taken for flow cytometry analyses were marked with a dashed line. The charts show representative results of three biological replicates carried out in duplicate in each replication. (c) Specific growth rates of fusion strains grown at 4°C and 37°C were calculated for exponentially growing cultures.

To develop an understanding of how the activity of *σ* ^B^ changes during growth and to investigate an effect of cold temperature on *σ* ^B^ activity, changes in *lmo2230*-promoter-driven fluorescence of* egfp*-tagged strains were analysed using flow cytometry at 4°C and at 37°C, at intervals of three days and 45 min, respectively (Figures 2(a) and 2(b)). As expected SGR values for fusion strains grown at 4°C were much lower than those recorded during growth at 37°C (Figure 2(c)).

**Figure 3:**
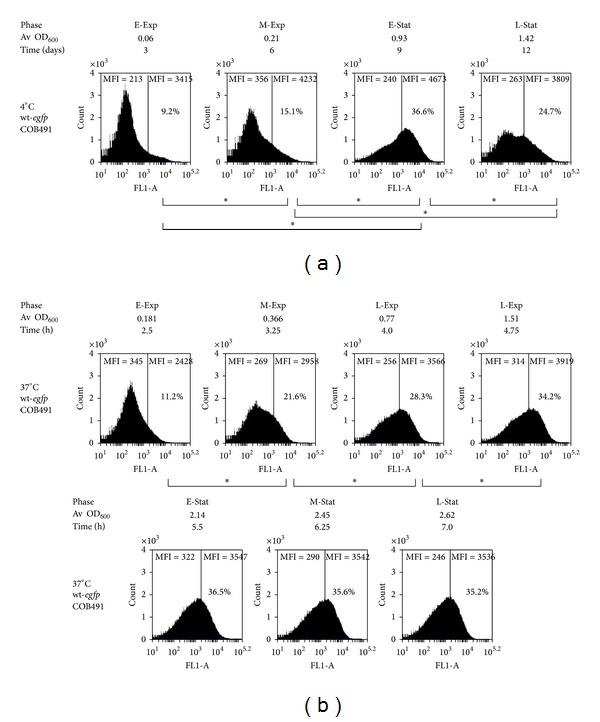
*σ* ^B^ is activated in *L. monocytogenes *EGD-e wild-type in growth phase dependent manner at 4°C and 37°C. Flow cytometry analyses of WT-*egfp* strain were performed with BD AcurriC6 at the interval of three days (a) and 45 min (b) at 4°C and 37°C, respectively, and data was processed with BD CFlow Software. The numbers in % indicate the proportion of cells within population with fluorescence above the highest autofluorenscence observed for parent wT strain. Mean fluorescence intensity (MFI) values were shown for autofluorescence range and for EGFP gate separately. Each sample was performed in biological triplicate with duplicate analyses in each replication and a minimum of 100,000 events recorded for each sample.

Flow cytometry of wt-*egfp* cells grown at 4°C showed that the fluorescent population increased during the exponential phase of growth from 9.2% to 15.1% in days 3 and 6, respectively (Figure 3(a)). The highest proportion of fluorescent cells was observed in early stationary phase, reaching 36.6% of the population. The level of EGFP-expressing cells within late stationary phase cultures dropped to 24.7% suggesting that *σ* ^B^ activity decreased slightly at 4°C during stationary phase. During growth at 37°C a very similar activation pattern was observed—in early exponential phase 11.2% of cells were found within EGFP-expressing gate while in mid-exponential phase 21.6% cells were recognised as fluorescent (Figure 3(b)). A further increase to 28.3% was observed in late exponential phase. During the stationary phase of growth at 37°C *σ* ^B^ activity did not increase at the rate observed when cells were dividing and the population of fluorescent cells remained stable with values around 35%. Based on the proportion of fluorescent cells within the population, revealed using flow cytometry of wt-*egfp* strain, a similar *σ* ^B^ activation pattern was observed both at 37°C and 4°C, with *σ* ^B^ activity increasing in the early exponential phase of growth and reaching a maximum level in stationary phase.

### 3.3. An Alternative Route of *σ* ^B^ Activation Exists at 4°C in the Absence of RsbV

**Figure 4:**
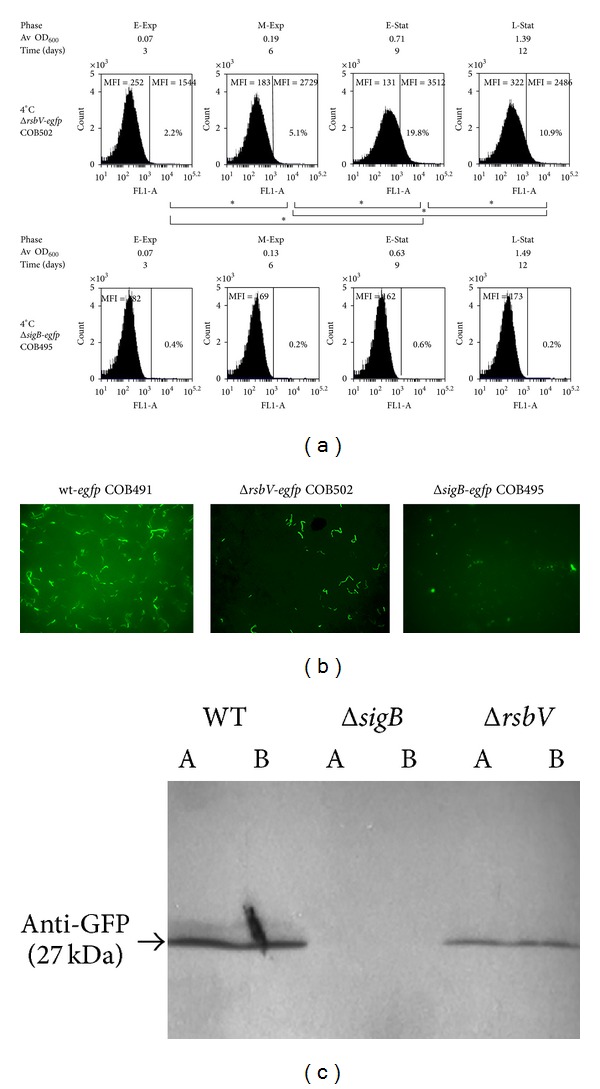
Activation of *σ* ^B^-dependent gene expression occurs in the absence of RsbV at 4°C. (a) Flow cytometry analyses of Δ*sigB*-*egfp* and Δ*rsbV*-*egfp* strains grown at 4°C were performed with BD AcurriC6 for cells taken at the interval of three days. The numbers in % indicate the proportion of cells within population with fluorescence above the highest autofluorenscence observed for parent WT strain. Mean fluorescence intensity (MFI) values were shown for autofluorescence range and for EGFP gate separately. Each sample was performed in biological triplicate with duplicate analyses in each replication and a minimum of 100,000 events recorded for each sample. (b) Microscopy of wT-*egfp, *Δ*sigB*-*egfp,* and Δ*rsbV*-*egfp* grown at 4°C and analysed in mid-exponential phase (day 6) revealed activation of *σ* ^B^ in the absence of RsbV. (c) Western blotting was carried out with anti-GFP antibody (Abcam) on SDS-PAGE separated protein extracts from stationary phase cells (day 12) of wT-*egfp, *Δ*sigB*-*egfp,* and Δ*rsbV*-*egfp* grown at 4°C. Each strain was analysed in biological duplicates ((a) and (b)).

To determine whether *σ* ^B^ activity observed in wt-*egfp* strain (Figure 3) depends solely on *σ* ^B^ and RsbV during growth, similar flow cytometry analyses were performed in Δ*rsbV* and Δ*sigB* backgrounds at 4°C and at 37°C at intervals of three days and 45 min, respectively (Figures 2(a) and 2(b); Supplementary material Figure S1, see Figure S1 in Supplementary Materials available on  line at http://dx.doi.org/10.1155/2014/641647). All *σ* ^B^ activity (i.e., EGFP-based fluorescence) was abolished in a Δ*sigB* background at both temperatures. Surprisingly, at 4°C cells of the Δ*rsbV-egfp *strain emitted fluorescence above the level of autofluorescence observed for the parent Δ*rsbV *strain (Figure 4(a)). In early exponential phase (day 3) 2.2% of population was recognised as EGFP-expressing while the proportion increased to 5.1% in late exponential phase (day 6). A further increase of the fluorescent population was observed in early stationary phase of growth where it reached its highest level of 19.8% (day 9) at 4°C. In late stationary phase (day 12) population of cells emitting fluorescence above autofluorescence background decreased to the level of 10.9%. In contrast to 4°C, none of the analysed populations of Δ*rsbV-egfp* or Δ*sigB-egfp* revealed significant fluorescence above the background at any stage of growth at 37°C (Supplementary material Figure S1), suggesting that all EGFP expression observed with flow cytometry in wt-*egfp* population at 37°C was both *σ* ^B^- and RsbV-dependent.

To evaluate the finding of RsbV-independent expression of EGFP at 4°C microscopic observations of wt*-egfp*, Δ*sigB-egfp* and Δ*rsbV-egfp* cells were performed. Both the wt*-egfp,* and Δ*rsbV-egfp* cultures contained fluorescent cells at 4°C, whereas none of the fields captured for a Δ*sigB-egfp* culture had fluorescent cells (Figure 4(b)). To rule out the EGFP expression in Δ*sigB *background at 4°C (noncell-shaped fluorescent particles), proteins were extracted from 100 mL of the fusion strains cultures grown to stationary phase (day 12) (Figure 4(c)). EGFP was detected using Western blotting with anti-GFP antibody in protein extracts from the wt*-egfp* and low but detectable levels were observed in Δ*rsbV-egfp, *while no evidence of EGFP in Δ*sigB-egfp* extracts was seen with Western blotting. These findings suggest that a low level of RsbV-independent *σ* ^B^ activity is present at 4°C, which is not seen at 37°C, perhaps suggesting an alternative route pathway for *σ* ^B^ activation at low temperatures. The data presented above indicate that *σ* ^B^-dependent transcription at 4°C can also occur in the absence of RsbV when, according to the present model, it is expected that *σ* ^B^ would be bound to RsbW and unavailable for transcription. One of the possible hypotheses suggested for a similar finding in *B. subtillis *was related to the instability of RsbW under nonoptimal temperature [27]. To determine whether RsbW is degraded or its levels are diminished at 4°C Western blot analyses were performed on protein extracts from stationary phase of growth with polyclonal antibodies raised in rabbits against RsbW from *L. monocytogenes* EGD-e strain. Similar patterns of anti-RsbW binding were observed in wt-*egfp* extracts from 37°C and 4°C suggesting that temperature has no effect on RsbW degradation (Supplementary material Figure S2). Furthermore, no changes in RsbW levels were recorded between wild-type and corresponding Δ*sigB* and Δ*rsbV* mutant strains grown at 4°C indicating that these deletions do not affect RsbW stability.

## 4. Discussion

### 4.1. *σ* ^B^ Activity Increases during Exponential Phase

The main aim of this study was to examine how physiological responses are coordinated in *L. monocytogenes* by the changes in the activity of an alternative sigma factor *σ* ^B^ during growth at 37°C and at 4°C. *σ* ^B^ activity was monitored by flow cytometry using cells expressing EGFP from strongly *σ* ^B^-dependent promoter (P_*lmo2230*_). The data show that within an exponentially growing population there is an increase in the proportion of cells displaying *σ* ^B^ activity and this continues to increase during growth, reaching maximal activation in stationary phase where ~35% of cells have fluorescence levels above background levels at both 37°C and 4°C. Previous reports showed limited activity of *σ* ^B^ in *L. monocytogenes* in exponential phase and the induction of *σ* ^B^-dependent expression during entry into stationary phase at 37°C [3, 28]. It was therefore somewhat unexpected to observe that the largest increase in the proportion of fluorescent cells in a population expressing EGFP from the *σ* ^B^-dependent promoter of *lmo2230* occurs during exponential phase, although maximal fluorescence occurs in stationary phase. Indeed this induction of *σ* ^B^ activity occurs in exponential phase at both 37°C and at 4°C. The mechanism triggering a reprogramming of the *σ* ^B^-dependent gene expression in exponential phase is unknown at present but could be related to increasing bacterial cell density. As the cells increase in number during growth the cells experience changes in the medium composition including depletion of some specific nutrients, reduced oxygen availability, medium acidification, accumulation of metabolites, and altered levels of signalling molecules. One or more of these changes could contribute to the activation of *σ* ^B^ during exponential growth and further experiments will be required to identify the specific signal involved.

### 4.2. *σ* ^B^ Activity Is Not Induced by Low Temperature in *L. monocytogenes*

In the present study a similar pattern of *σ* ^B^ activation was observed within populations grown in cold (4°C) or optimal (37°C) temperatures. At both temperatures *σ* ^B^ activity was found to be increased during exponential phase, reaching maximal levels of activity early in stationary phase (Figures 3(a) and 3(b)). These data suggest that *σ* ^B^ may not play a central role during cold adaptation in *L. monocytogenes*. This conclusion is consistent with the findings of a number of proteomic and transcriptomic studies that have sought to define the *σ* ^B^ regulon and the cold stimulon in this pathogen. Only a very limited number of genes belonging to the *σ* ^B^ regulon [7, 9, 29] are also found to be present in the cold stimulon [30–32]. Of the 30 genes listed as being upregulated in both exponential and stationary phase during growth at 4°C compared to 37°C [31] only two (*lmo1670* and *lmo1937*) were described before as members of *σ* ^B^ regulon in one study [29] but not mentioned elsewhere. Furthermore, in the present study Δ*sigB*-*egfp *and Δ*rsbV*-*egfp* strains displayed comparable growth rates to the wild-type-*egfp*, demonstrating that *σ* ^B^ activity is not critical for growth of *L. monocytogenes* EGD-e at low temperature in BHI. This is a conclusion that Chan and colleagues also highlighted in an earlier study [17]. Taken together these results suggest that the modulation of *σ* ^B^ activity that occurs during growth at 4°C is primarily influenced by the growth phase rather than the growth temperature.

### 4.3. RsbV-Independent *σ* ^B^ Activation

Although the high degree of conservation between the *sigB* operons in *B. subtilis* and *L. monocytogenes* suggests that regulation of *σ* ^B^ is similar in both organisms [12, 33], this assumption has not yet been rigorously tested in *L. monocytogenes*. Initial studies on the *L. monocytogenes* system suggest that there may be fundamental differences in how *σ* ^B^ is activated in this pathogen. Firstly, environmental (physicochemical) stress and energy stress both act via RsbT in *L. monocytogenes *[14], whereas energy stress is sensed in an RsbT-independent manner in *B. subtilis* [34]. More recently it has been shown that in *L. monocytogenes* energy stress signals can also influence *σ* ^B^ activity independently of both RsbT and RsbU [13].

Many features of the upstream Rsb-dependent model regulating *σ* ^B^ activity remain unclear in *L. monocytogenes*. Chaturongakul et al. [14] reported that RsbT contributes to *σ* ^B^ activation through RsbV during exposure to both environmental and energy stresses in *L. monocytogenes* while in *B*. subtillis two separate pathways exist [34]. A more recent study with the opuCA-lacZ reporter suggests that in *L. monocytogenes σ* ^B^ induction after energy stress enters the network through an RsbT-independent pathway and that RsbU does not modulate that response [13]. The *σ* ^B^ regulon was not shown to be induced by any of the stimuli described; thus far in Δ*rsbV *background and both Δ*rsbT* and Δ*rsbV* showed survival reductions similar to those of the Δ*sigB* strain at optimal temperature [35]. Thus, it was surprising to note that *σ* ^B^-dependent expression occurs at 4°C in Δ*rsbV* background when *σ* ^B^ is expected to be completely inactivated by RsbW. RsbV-independent activation of *σ* ^B^ has been reported for chill-stressed *B. subtilis* and it was also shown to be RsbU and RsbP independent [36]. In contrast, RsbT and RsbU were shown to be required for *σ* ^B^ induction in response to cold downshift from 37°C to 7°C in *L. monocytogenes* [13]. Changes in the RsbW : SigB ratio at 4°C or possible chill-induced changes in RsbW functionality might contribute to the observed RsbV-independent effects on *σ* ^B^ activity in the present study, but further experiments will be required to clarify this point.

In conclusion, the present study shows that *L. monocytogenes* cells induce *σ* ^B^ activity in early exponential phase and *σ* ^B^ is maximally active in the population entering the stationary phase of growth. Moreover, a similar pattern of *σ* ^B^ activation is observed within populations grown in the cold and at optimal temperatures suggesting that *σ* ^B^ does not play a pivotal role in cold adaptation. Finally, we demonstrate that *σ* ^B^ activation can occur independently of the antisigma factor antagonist RsbV in chill-stressed cells, but the mechanism underpinning this effect requires further investigation.

## Supplementary Material

Figure S1 shows that influence of growth phase on EGFP expression at 37°C is dependent on **σ** ^B^ and RsbV.
Figure S2 indicates that RsbW stability is unaffected by growth temperature or *rsbV* genotype.

## Figures and Tables

**Table 1:** Plasmids and strains used in this study.

Plasmid	Abbreviated name	Source or reference	Collection number
Champion pET101 directional TOPO expression vector containing topoisomerase for efficient and simple cloning	pET101D	Invitrogen	N/A
Champion pET101 directional TOPO expression vector with wild type *rsbW* with N-terminal His_6_-tag	pEC02	This study	N/A
Champion pET101 directional TOPO expression vector with wild type *sigB* with N-terminal His_6_-tag	pEC03	This study	N/A

Strains			
One shot TOP10 chemically competent *E. coli* F^−^ * mcr*A Δ(*mmr-hsd*RMA-*mcr*BC) Φ80*lac*ZΔM15 Δ*lac*X74 *rec*A1 *ara*D139 Δ(*ara-leu*)7697 *gal*U *gal*K *rps*L (Str^R^) *end*A1 *nup*G	*E. coli* TOP10	Invitrogen	N/A
BL21 Star (DE3) one shot chemically Competent *E. coli* F^−^ * imp*T *hsd*S_B_ (r_B_^−^m_B_^−^) *gal dcm rne*131 (DE3)	*E. coli *BL21 (DE3)	Invitrogen	N/A
BL21 Star (DE3) *E. coli*/pEC02	*E. coli/rsbW-his6 *	This study	COB275
BL21 Star (DE3) *E. coli*/pEC03	*E. coli/sigB-his6 *	This study	COB276
*L. monocytogenes* EGD-e	wt	K. Boor	COB261
*L. monocytogenes* EGD-e Δ *sigB *	Δ*sigB *	K. Boor	COB262
*L. monocytogenes* EGD-e Δ *rsbV *	Δ*rsbV *	Utratna et al. 2012 [24]	COB411
*L. monocytogenes *EGD-e::pKSV7-P_*lmo*2230_::*egfp *	wt-*egfp *	Utratna et al. 2012 [24]	COB491
*L. monocytogenes *EGD-e Δ*sigB*::pKSV7-P_*lmo*2230_::*egfp *	Δ*sigB*-*egfp *	Utratna et al. 2012 [24]	COB495
*L. monocytogenes *EGD-e Δ*rsbV*::pKSV7-P_*lmo*2230_::*egfp *	Δ*rsbV*-*egfp *	Utratna et al. 2012 [24]	COB501

**Table 2:** Primers used in this study.

Oligo	Primer name	Primer sequence (5′ to 3′ direction)	Source
COB330	*rsbW*-CACC Fwd	CACCATGGCAACAATGCATGACAAAATTAC	This study
COB331	*rsbW- h* *is*_6_ Rev	TCAGTGGTGGTGGATGATGATGGGTTGAGATACTTTTGGC	This study
COB332	*sigB*-CACC Fwd	CACCATGCCAAAAGTATCTCAACCTG	This study
COB333	*sigB- h* *is*_6_ Rev	TTAATGGTGATGGTGATGGTGCTCCACTTCCTCATTCTG	This study

**Table 3:** Antibodies used in this study.

Antibodies	Dilution	Tween20 concentration (v/v)	Source
1°Ab polyclonal rabbit anti-SigB	1 : 2,000	0.5%	This study
1°Ab polyclonal rabbit anti-RsbW	1 : 1,000	0.1%	This study
1°Ab polyclonal chicken anti-OpuCA	1 : 2,000	1.0%	Utratna et al. 2011 [22]
1°Ab polyclonal chicken anti-GFP	1 : 2,000	0.5%	Abcam
2°Ab HRP-conjugated Goat anti-rabbit	1 : 20,000	0.5% or 0.1%	Promega
2°Ab HRP-conjugated Goat anti-chicken	1 : 20,000	1.0% or 0.5%	Promega
