# Supplementary material for: Effects of Growth Phase and Temperature on σ B Activity within a Listeria monocytogenes Population: Evidence for RsbV-Independent Activation of σ B at Refrigeration Temperatures
Source: Biomed Res Int. 2014 Mar 5;2014:641647. doi: 10.1155/2014/641647 (PMC3964741; doi:10.1155/2014/641647)
Supplement: Supplementary file 1 — Figure S1 shows that influence of growth phase on EGFP expression at 37°C is dependent on σ B and RsbV. Figure S2 indicates that RsbW stability is unaffected by growth temperature or rsbV genotype. [file 641647.f1.zip › Description.docx]

**Supplementary Material**

**Figure S1 shows that influence of growth phase on EGFP expression at 37°C is dependent on σ^B^ and RsbV.** Flow cytometry analyses of (A) Δ*rsbV*-*egfp* and (B) Δ*sigB*-*egfp* cultures grown at 37°C were performed with BD AcurriC6 for cells taken at suitable intervals during growth. The numbers in % indicate the proportion of cells within population with fluorescence above the highest autofluorenscence observed for parent WT strain (*egfp*^-^). Mean fluorescence intensity (MFI) values were shown for autofluorescence range and for EGFP gate separately. Each sample was performed in biological triplicate with duplicate analyses in each replication and a minimum of 100,000 events recorded for each sample.

**Figure S2 indicates that RsbW stability is unaffected by growth temperature or *rsbV* genotype.** Western blot analysis of RsbW levels were carried out for cultures grown at either 4°C or 37°C. Samples were prepared from P*lmo2230-egfp* derivatives of either the wild-type (WT), the ∆*sigB* mutant (∆*sigB*), or the mutant lacking *rsbV* (∆*rsbV*). Polyclonal antibodies (1:1,000 dilutions) against the purified RsbW protein were used in the Western blot analysis. The position of RsbW was assigned on the blots based on the predicted molecular mass of the RsbW protein (17 KDa). Higher molecular weight bands are likely to represent no-specific binding of the antibodies.
