# Supplementary figures and images for: Effects of Growth Phase and Temperature on σ B Activity within a Listeria monocytogenes Population: Evidence for RsbV-Independent Activation of σ B at Refrigeration Temperatures
Source: Biomed Res Int. 2014 Mar 5;2014:641647. doi: 10.1155/2014/641647 (PMC3964741; doi:10.1155/2014/641647)

Fig. S1

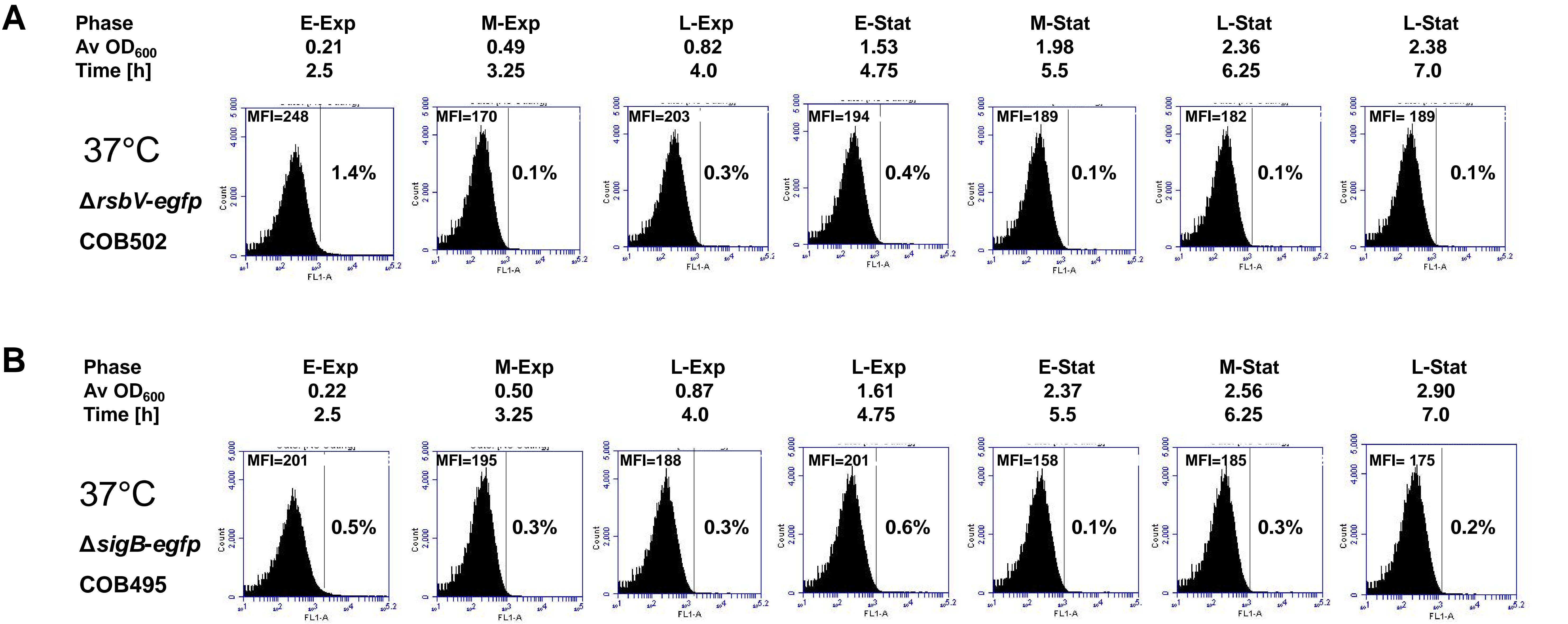

Fig. S2

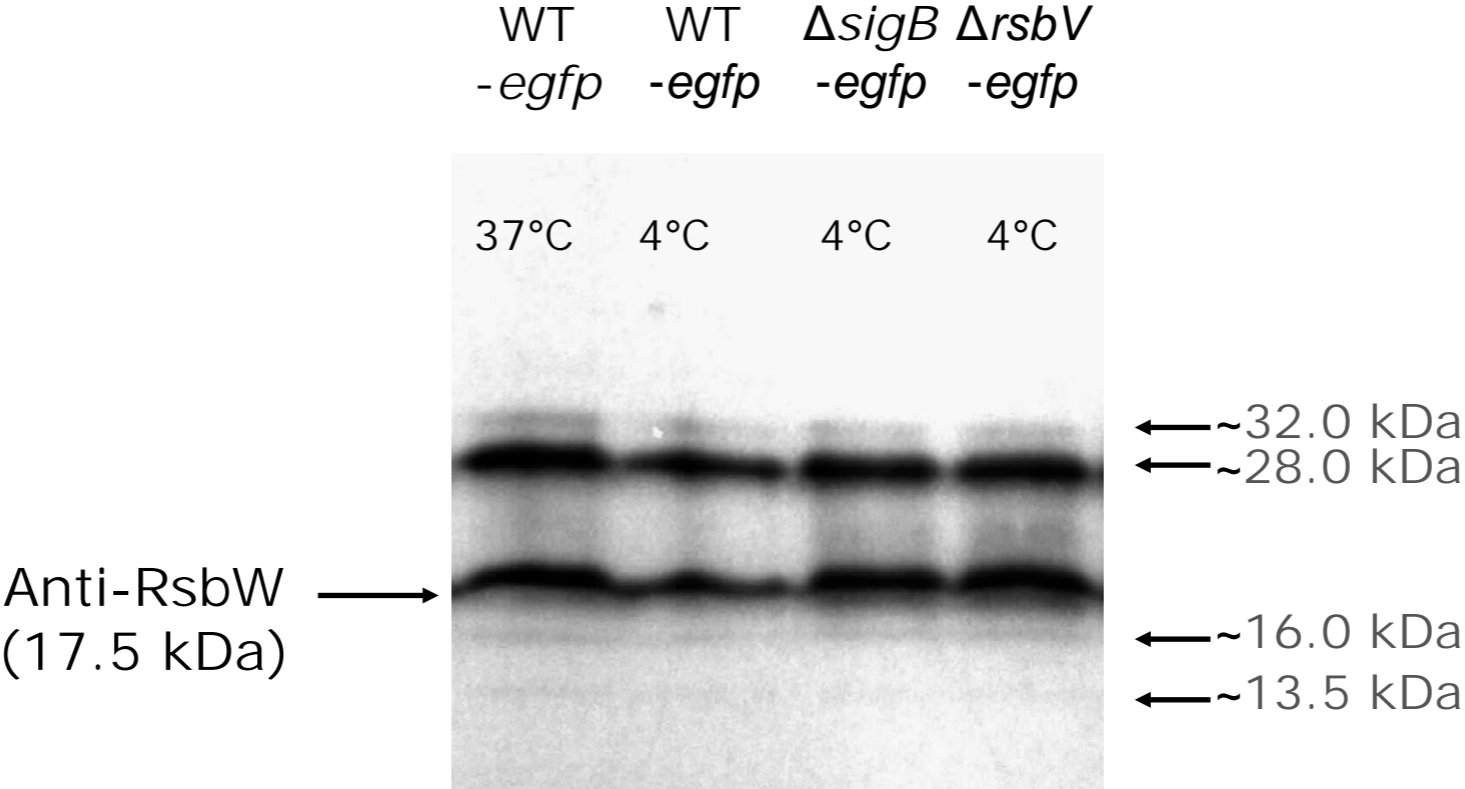

Supplement: Supplementary file 1 — Figure S1 shows that influence of growth phase on EGFP expression at 37°C is dependent on σ B and RsbV. Figure S2 indicates that RsbW stability is unaffected by growth temperature or rsbV genotype. [file 641647.f1.zip › Suppl Figures.pdf]
